# Supplementary figures and images for: HERVH-derived lncRNAs negatively regulate chromatin targeting and remodeling mediated by CHD7
Source: Life Sci Alliance. 2021 Oct 18;5(1):e202101127. doi: 10.26508/lsa.202101127 (PMC8548210; doi:10.26508/lsa.202101127)

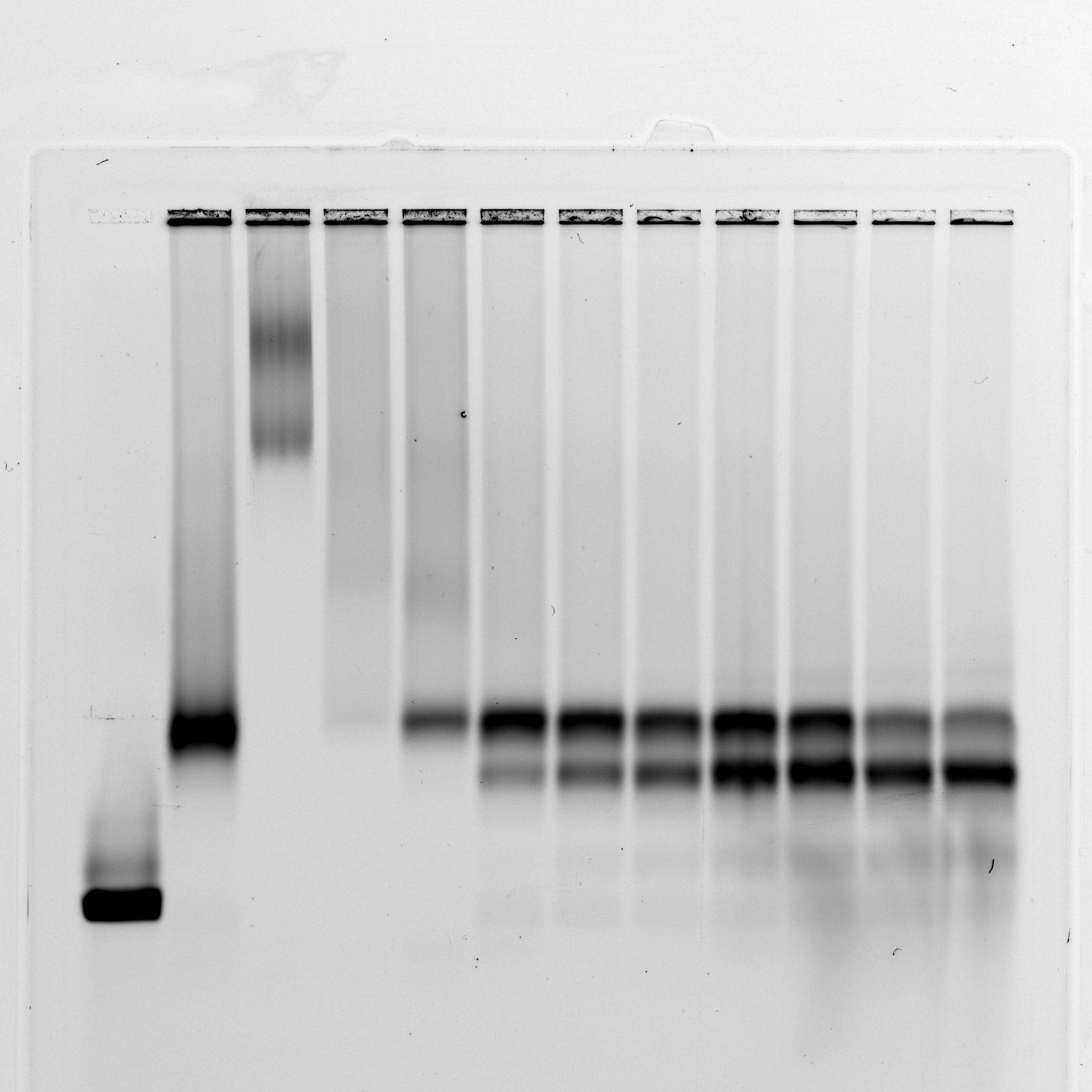

Supplement: Supplementary file 2 [file LSA-2021-01127_SdataF5.tif]
